# Bacteriophage Mediated Killing of *Staphylococcus aureus* In Vitro on Orthopaedic K Wires in Presence of Linezolid Prevents Implant Colonization

**DOI:** 10.1371/journal.pone.0090411

**Published:** 2014-03-03

**Authors:** Sandeep Kaur, Kusum Harjai, Sanjay Chhibber

**Affiliations:** Department of Microbiology, Panjab University, Chandigarh, India; University Hospital Münster, Germany

## Abstract

**Background:**

Infections of bone and joint tissues following arthroplasty surgeries remain a major challenge in orthopaedic settings. Methicillin resistant *Staphylococcus aureus* (MRSA) is recognised as an established pathogen in such infections. Combination therapy using linezolid and bacteriophage impregnated in biopolymer was investigated in the present study as an alternative strategy to prevent MRSA colonisation on the orthopaedic implant surface.

**Methodology:**

Coating of stainless steel orthopaedic grade K-wires was achieved using hydroxypropylmethlycellulose (HPMC) mixed with phage alone, linezolid alone and phage and linezolid together. The potential of these agents to inhibit adhesion of *S.aureus* (MRSA) 43300 on K-wires was assessed. Coated and naked wires were analysed by scanning electron microscopy (SEM) and fluorescent staining.

**Result:**

Significant reduction in bacterial adhesion was achieved on phage/linezolid wires in comparison to naked as well as HPMC coated wires. However, maximum reduction in bacterial adherence (∼4 log cycles) was observed on the wires coated with phage-linezolid combination. The frequency of emergence of resistant mutants was also negligible in presence of both the agents.

**Conclusion:**

This study provides evidence to confirm that local delivery system employing linezolid (a potent protein synthesis inhibitor) along with a broad spectrum lytic bacteriophage (capable of self-multiplication) is able to attack the adhered as well as surrounding bacteria present near the implant site. Unlike other antibiotic based therapies, this combination has the potential to significantly restrict the emergence of resistant mutants, thus paving the way for effective treatment of MRSA associated infection of medical implants.

## Introduction

Orthopaedic devices used in joint and hip replacement surgeries have greatly improved the health outcomes for patients. However, implant associated infections remain a major problem in hospital settings [1], [2].Treatment of the open fracture exposes the sterile body sites to the external environment that leads to introduction of the pathogen either on the implant itself or into the surgical site [3].*Staphylococcus aureus* is a major pathogen involved in orthopaedic implant infections. After adherence, it tends to form biofilm on the implant surface, which acts as a barrier for the penetration of antibacterial compounds and host's immune system [4]–[6]. Bacteria within the biofilms are more recalcitrant to the action of antibiotics. To worsen the scenario, prevalence of methicillin resistant *S.aureus* (MRSA) is particularly alarming in orthopaedic trauma and joint replacement patients [7]–[9].This makes the empherical therapy of implant infections, particularly those infected with MRSA, a bigger challenge with limited choice of antibiotics.

An alternative approach is the local application of antibiotics directly at the site of infection by means of an adequate carrier or by placing polymer coated implants (stainless steel or titanium implants) loaded with antimicrobial agents [10]. Newer systems use biodegradable implant coatings to facilitate the controlled release of antibiotic [11]–[14], at a higher concentration and over a longer period of time. This approach may particularly be beneficial in situations where concentration of antibiotic required is 10–20 times higher than MIC is required to remove the adherent bacteria. Gentamicin, tobramycin and also vancomycin impregnated into various biopolymers are primarily used to coat the orthopaedic implants for their local delivery. However, resistance to aminoglycoside among MRSA isolates is now common. Although there are few reports of emergence of vancomycin intermediate-resistant *S. aureus* (VISA) and vancomycin-resistant *S. aureus* (VRSA), but the rising MICs of vancomycin among vancomycin susceptible *S. aureus* isolates, also referred to as “vancomycin creep”, further suggests the need for newer agents to tackle MRSA threat in near future [15]–[17].

With controlled release systems, the antibiotic levels fall to sub-therapeutic levels, and as the levels drop to sub-inhibitory concentrations, the risk of developing resistance among bacteria gets higher [18], [19].Newer approaches include covalent attachment of antibiotics or metal ions to implant surfaces. These tethered antimicrobial agents represent a favourable option because the antibiotics are firmly attached and no bulk tissue toxicity is anticipated [20].However, frequency of occurrence of resistance in such situation has not been studied so far. Also, tethered antibiotic is only active in the space immediately adjacent to the implant and thus fails to act on the surrounding bacteria. Silver impregnated devices have also shown encouraging antimicrobial properties *in vitro* but the efficacy is still not clinically proven. Also, silver toxicity can occur at serum levels as low as 0.3 µg/mL with argyria, leukopenia, alterations in renal, hepatic, and neural tissues being reported [21], [22]. These problems call for delivery systems involving safer antibacterial agents that do not suffer from the problem of emergence of resistance as well as are potent enough to tackle the adherent as well as surrounding bacteria.

In the present manuscript, lytic *S.aureus* phage has been used in combination with linezolid in a biodegradable drug delivery system for the controlled release of the two agents at the implant site. Phages, are good candidates to treat orthopaedic infections because of three strong reasons. Firstly, from a clinical standpoint, phage therapy is safe with no reports of any adverse effects or local tissue toxicity. They do not affect eukaryotic cells even at higher concentrations. [23]–[26]. Secondly, phages possess self-reproducing ability, giving them an advantage over the other antibiotic based delivery system [24], [27]. They are able to multiply *in vivo* in animal tissues as long as the corresponding host bacterium is present. This phenomenon is referred to as “auto-dosing” whereby phages themselves by exponential growth contribute to establishing high phage titers [28].Thirdly, phage production is simple and inexpensive [29], [30]. Linezolid was selected for treatment as it holds the advantage of possessing 100% oral bioavailability, favourable pharmacokinetic profile and better penetration into osteo-articular tissue [31]–[33]. Evidence also suggests that linezolid efficiently checks the emergence of resistant mutants when used in combination with other drugs [34]. Linezolid also possesses the ability to suppress toxin production in *S.aureus*, thus improving the clinical outcome [35]–[37]. This combination approach has not been explored in the fact for treating MRSA mediated orthopaedic infections. It shall deliver the two agents directly at the implant site providing an aggressive approach at the initial stage itself, preventing initial adherence and seeding. The use of these two agents (with entirely different mode of action) together shall keep a check on the frequency of emergence of resistance as well.

## Materials and Methods

### Bacterial strains and Bacteriophage used

*S. aureus* ATCC 43300 (methicillin resistant *S.aureus* [MRSA]) and *S.aureus* ATCC 29213 (methicillin sensitive *S.aureus* [MSSA]) from ATCC, Mannasse, USA were used in this study. Clinical isolates of *S. aureus* were procured from Post-graduate Institute of Medical Education and Research (PGIMER), Chandigarh, India. The strains were isolated from clinical specimens (nasal screening swabs, blood, pus, soft tissue, wound swabs, respiratory samples and body fluids) from both in-patient as well as out-patients from in as well as around places near Chandigarh, India.

These strains were identified on the basis of Gram reaction, growth on mannitol salt agar (MSA), catalase activity, and coagulase test. Methicillin resistance was determined using cefoxitin disk on Mueller-Hinton agar (Oxoid) followed by determination of MICs of oxacillin for these strains as recommended by Clinical and Laboratory Standards Institute (CLSI) [38].A total of forty five MRSA isolates were selected and stored in glycerol at −80°C. These strains were used for determining the lytic spectrum/host range of the isolated phage. *S. aureus s*pecific lytic bacteriophage, MR-5 with broad host range (active against both MRSA as well as MSSA strains), belonging to family *Myoviridae*, isolated and characterized in our laboratory earlier was used in the present study [39].

### Polymer and Implants used

Hydroxypropylmethylcellulose (HPMC) powder K4MP grade was obtained from Dow Chemicals, Michigan, IL, USA. This polymer was chosen because of its proven biocompatibility with phages, previously standardized in our laboratory [40].Commercially available orthopaedic grade Kirschner-wires (K-wires) of stainless steel (diameter 1.5 mm) were procured from the local market and cut into 30 mm length, cleaned and autoclaved.

### Preparation of purified MR-5 phage

High-titer MR-5 suspension was prepared according to the method described by Langley *et al.* [41]. 400 ml of BHI broth inoculated with overnight host culture and phage was allowed to incubate till lysis occurred with complete clearance of broth. The broth was then centrifuged and the supernatant containing MR-5 was collected and passed through a 0.22-µm-pore-size filter. The filtrate was subjected to concentration using Millipore Labscale TFF system (Pellicon) and the 10 K (polyethersulfone) membranes for a period of 3–4 hours till the sample volume reduced from initial 400 ml to 25–30 ml. The concentrated product was incubated with DNase I (0.25 mg/ml) for 1 h at 37°C to digest the genomic DNA. NaCl (final concentration of 1M) and polyethylene glycol (PEG) 8000 were added to the concentrated lysate and kept at 4°C overnight. Next day, the precipitate was collected by centrifugation, it was dialyzed against phosphate-buffered saline (PBS) overnight at 4°C, finally passed through a 0.22-µm-pore-size filter and stored at 4°C till further use. The final product so obtained was subjected to phage titration according to the modified double layer agar (DLA) technique [39].A high titer stock obtained with a final phage titer of 1.5×10^9^ PFU/ml (stored in aliquots) at 4°C was used in all the experiments.

### Preparation of hydrogel

HPMC gel formulations were prepared as described by Alfadhel *et al.* [42] by dissolving the required amount of HPMC powder in one third of the final volume of sterile distilled water at 80–90°C.HPMC solution was added slowly with constant stirring until a consistent dispersion was obtained. The remaining amount of sterile water was then added (using water at room temperature) with constant stirring to obtain a uniform gel suspension of 5 ml as final gel volume. The gel was stored at 4°C overnight to partially degas the gel and allow complete hydration of the polymer chains. The double strength gels (2X) were prepared so that final concentration of HPMC after addition of phage or antibiotic solution was 2,4 and 6% respectively.

### HPMC coating formulations

Three different coating formulations were prepared for the study:

1. Phage mixed with HPMC gel denoted as **H-P:** For preparing **H-P** formulation, high titer phage stock solution containing a final titer of 1.5×10^9^ PFU/ml was added to HPMC gel (1:1) and stirred gently for uniform distribution in the gel matrix.
2. Linezolid mixed with HPMC gel denoted as **H-L:** For preparing **H-L** formulation, stock solution of linezolid was first prepared in DMSO and later, working stock was prepared in sterile distilled water. A required volume of the working stock was added to the gel to obtain a final drug concentration of 5% (w/w).

[Note: H-P and H-L formulation with all three concentration of HPMC gel (2,4 and 6%) were prepared i.e in total six formulations were prepared].

1. Phage as well as Linezolid mixed with HPMC gel denoted as **H-P-L:** For preparing H-P-L formulation, both the phage suspension as well as linezolid working solution were added simultaneously with constant stirring to obtain a uniform gel suspension. However, the formulation containing linezolid and phage impregnated in the coating solution [H-L-P] was prepared in 4% HPMC only. This concentration was selected based on the elution kinetics of both phage and linezolid described in later sections.

All the coating formulations were stored at 4°C till further use.

### Polymer coating

Implants were coated with HPMC (2%, 4%, 6% w/v) as per the method of Gollwitzer *et al*. [43]. Approximately, 30 mm K-wires were autoclaved and used for coating. Wires were dip-coated in 5 ml of coating formulations. All coating steps were carried out under aseptic conditions and the K-wires were dried in laminar air flow. The coating procedure was repeated four times to increase the linezolid and/or phage content on each K-wire. Each wire was inspected visually after coating for uniform dispersion of gel matrix.

The coating on K-wires coated with 6% HPMC coating formulations was visually uneven and was thus was not chosen for further experiments. Thus, coated wires with 2 and 4% coating formulation were used in further experiments.

### Elution Assay

In order to determine the rate of release of phage and antibiotic from the implant surface, the coated K-wire was placed in tubes each containing 1 ml of PBS (one wire per tube). After 30 min of immersion the wire was taken out and passed to fresh microfuge tube containing 1 ml fresh PBS. The previous PBS solution was processed for determining the titer of phage and linezolid released. The same process was repeated at different time points (1 h, 2 h, 3 h, 4 h, 8 h, 24 h, and 48 h, 120 and 168 h) with K-wires being passed into fresh PBS each time. The wires (n = 6) having either H-P or H-L were processed at each time point. Rate of phage release at each time point was determined by titrating each aliquot according to the modified double layer agar (DLA) technique. Linezolid concentration was determined by employing two different methods:

- **Bioassay.** This assay was performed as per the method of Wiederhold *et al*. [44].In brief, Mueller Hinton agar plates were flooded with a standardized cell suspension (1×10^4^ CFU/mL) of *S.aureus* ATCC 29213 and allowed to dry at ambient temperature. Wells were bored into the agar and 100 µL aliquots of standardized linezolid solution (0.25–64 µg/ml) or unknown sample were pipetted into the wells. The plates were incubated for 24 h at 37°C and inhibition zones measured following incubation. Each bioassay was performed in triplicate. A standard curve was plotted from which the unknown concentration of linezolid in the test sample was determined.
- **Colorimetric assay.** This assay was performed as per the method described by Patel *et al*. [45]. In brief, 0.5 ml of standard linezolid solution (0.5–200 µg/ml) or unknown sample was mixed with 0.5 ml of Follins - Ciocalteus (F-C) reagent. This was followed by addition of 1 ml of 20% sodium carbonate solution. The mixture was incubated for 15 minutes at room temperature and absorbance was measured at 750 nm (Shimadzu model 1900 double beam UV/visible spectrophotometer).The concentration of the test sample was determined from the standard curve.

### Phage stability studies

The stability of phage in the chosen HPMC coating formulation (4%) was studied over a period of 20 days at room temperature. Also, phage stability on the HPMC-phage coated wires was tested on different days. For this, at each time point three phage coated K-wires were immersed in three different tube, each containing 1 ml PBS solution and incubated for 24 hours at room temperature. The PBS suspension was later centrifuged at 10,000 rpm/15 min and the clear supernatant was subjected to phage titration giving the total phage titer expressed in terms of PFU/ml.

### Bacterial adherence

Adhesion of viable bacteria was evaluated in a bacterial adhesion assay as described by McMillan *et al*.[3]. *S.aureus* 43300 (ATCC, MRSA) was grown overnight in brain heart infusion (BHI) broth. Next day, the cells were pelleted, washed twice and finally suspended in PBS (pH 7.4) to obtain a cell density of 10^7^ CFU/ml. Coated (HPMC control, H-P, H-L, H-L-P) and naked K-wires (n = 4 per representative group) were immersed in 2 mL of this bacterial suspension. One set of wires was incubated for 6 h and the other set of wires for 24 h at 37°C under static conditions. After washing in normal saline, the K-wires were placed in vials containing 2 mL of trypsin solution (1% w/w) and sonicated for 15 min to remove the adhering microorganisms. Following sonication, each of the serially diluted trypsin treated preparation was plated on nutrient agar plates for the quantification of viable organisms. For each set of experiments, the percentage of CFU recovered from different (H-P, H-L and H-L-P) groups was compared with the average CFU obtained from the naked and HPMC control [H] groups.

### Visualization of bacterial adherence on K-wires

In an attempt to visualise adhered bacteria on the K-wires, two different fluorescent dyes were used to stain the adhered bacteria on the naked as well as HPMC coated wires.The naked as well as HPMC coated wires were incubated in presence of 10^7^ CFU/ml of *S.aureus* 43300 for 24 h. The wires were then washed twice with PBS, pH 7.4.The washed wires were dipped in solution of Syto9 and Propidium iodide (part of the BacLight Viability assay kit; Molecular Probes, Invitrogen, Germany) for 15 minutes in dark and allowed to dry. Syto 9 stains the live cells whereas PI stains the dead cells adhered on the wires. Naked wires as well as HPMC wires without any adhered bacteria were also stained in similar way and acted as control. All the stained wires were visualized under epi-fluorescence microscope (Nikon Eclipse 80i).

Presence of polymer coating as well as adhered bacteria after 24 h of immersion in BHI broth with 10^7^ CFU/ml of *S.aureus* 43300 on K-wires was visualized by scanning electron microscopy (SEM). Electron micrographs were taken at 25 kV using a JEOL JSM-6100 scanning electron microscope by the gold sputtering method using fine coat ion sputter, JFC-1100.

### Appearance of bacteriophage insensitive mutant (BIM) and linezolid resistant mutants

The frequency of spontaneous mutation in *S.aureus* 43300 on exposure to phage and linezolid was determined. For BIM frequency, plaque assay was performed using an overnight culture of *S.aureus* 43300 containing known cell numbers and phage added at a MOI of 1 and 10 respectively. The plates were incubated overnight at 37°C. All resulting colonies were counted, and the BIM frequency was determined by dividing the number of surviving colonies by the original bacterial titer. Similarly, spontaneous mutation frequency for linezolid was also determined at 8 µg/ml according to the method of O'Neill *et al.* [46] using cation adjusted Mueller Hinton agar plates. The frequency was determined by dividing the number of surviving colonies on selective plates with total number of colonies on non-selective plates after 48 hours of incubation.

Frequency of appearance of resistant mutants in presence of both phage and linezolid together was determined by performing the plaque assay on selective plates with 8 µg/ml of linezolid and with phage added at MOI -1 as well as 10.

### Statistical Methods

All the data is expressed as mean ± standard deviation of replicated values (n = 4) where indicated. The statistical significance of differences between groups was determined by the Student's t-test (two groups), One-way ANOVA followed by a Tukey test using Sigma Stat, Graph pad prism (Graph pad software, San Diego, CA) was used. p value of less than 0.05 and 0.01 was considered significant and highly significant respectively.

## Results

### Elution assay

Phage elution was studied over a period of seven days from the coated wires loaded with two different HPMC concentrations (2 and 4% w/v).The results in Table 1 show the number of phages released (in terms of PFU/ml) from the coated wires at different time points. Maximum phage particles elution after 24 hours was seen from the wires coated with 4% HPMC. There was a drop from an initial titer of 10^9^ PFU/ml to 1.12×10^6^ PFU/ml. This indicated a significant loss of phage particles during processing and coating steps itself. In case of 2% HPMC gel, majority of phages were released in the first hour in PBS, after which there was gradual but steady release from the coated surface. Phage release continued till 2 days post-immersion with 2.1×10^2^ PFU/ml additional phages detected at 48 hours. However, no phage release was seen beyond this time point. On the contrary, in case of higher viscosity gel 4% (w/v), phages were released steadily in small bursts over a longer period of time. The number of phage particles eluted was also comparatively higher than that released from 2% coated wires. Maximum release of phage occurred between 8^th^ and 24^th^ hour of sampling with 11.2×10^5^ phages being detected during this period. Elution continued till 96 hour with no phage particles seen beyond this time point. Finally, 4% HPMC concentration was selected because it showed steady elution of phages at a higher concentration over a longer period of time into the surrounding medium.

**Table 1:** Elution of phage from HPMC coated wires at different time points.

Time (hours)	Phage elution (PFU/ml) from 2% HPMC coated wires	Phage elution (PFU/ml) from 4% HPMC coated wires
1	(1.9±0.06) ×10^5^	(1.87±0.07) ×10^5^
2	(2.1±0.21) ×10^4^	(1.23±0.101) ×10^5^
4	(3.2±0.18) ×10^4^	(1.5±0.15) ×10^5^
6	(2±0.11) ×10^4^	(2.2+0.11) ×10^5^
8	(1.8±0.08) ×10^4^	(2.1±0.12) ×10^5^
24	(3±0.17) ×10^4^	(1.12±0.05) ×10^6^
48	(2.1±0.14) ×10^3^	(2.3±0.17) ×10^4^
96	NE	(1.1±0.21) ×10^2^
120	NE	NE
168	NE	NE

Each value represents mean ± S.D of six independent values obtained from.

n = 6 wires at each time point. NE: no further elution.

### Linezolid Elution Kinetics

Elution of linezolid from HPMC coated wires was studied by employing two different methods. Almost similar pattern of drug release was observed (Table 2) at different time points with either of the two methods. In case of 2% HPMC gel coated wires, gradual elution of linezolid continued till 48 h, giving a total concentration of ∼16 µg/ml at 48 h. Significant elution (∼7–8 µg/ml) of linezolid was seen in the first 30 minutes of immersion. The rest of the drug was steadily eluted in the first 8 h of immersion. In between 8 and 24 h, there was a release of ∼3 µg/ml of additional drug from the coated matrix. However, after 96 h, no major elution of linezolid occurred.

**Table 2:** Elution of linezolid (µg/ml) from the K-wires coated with either 2% or 4% HPMC containing 5%(w/v) of linezolid as determined by colorimetric/bioassay.

Time(h)	Linezolid eluted from 2% HPMC+5%(w/v) Linezolid -Colorimetric assay	Linezolid eluted from 2% HPMC+5%(w/v)Linezolid -Bioassay	Linezolid eluted from 4% HPMC+5%(w/v)Linezolid -Colorimetric assay	Linezolid eluted from 4% HPMC+5%(w/v)Linezolid -Bioassay
0.5	7.17±0.14	8.13±0.17	9.11±0.14	8.43±0.17
1	1.26±0.05 (8.43)	1.01±0.11 (9.14)	2.7±0.11 (11.8)	2.27±0.08 (10.7)
2	0.69±0.04 (9.12)	0.73±0.03 (9.87)	1.32±0.04 (13.12)	1.4 (12.1)
4	1.09±0.1 (10.21)	0.97±0.04(10.84)	1.28±0.10 (14.4)	1.77 (13.87)
6	1±0.11 (11.22)	0.80±0.03 (11.64)	2.31±0.07 (16.71)	1.27 (15.14)
8	0.8±0.07(12.01)	0.67±0.06 (12.31)	1.99±0.12 (18.7)	1.98 (17.12)
24	2.13±0.11 (14.14)	3.16±0.17(14.8)	6.39±0.21 (25.1)	5.28±0.20(22.4)
48	0.84±0.03 (14.98)	0.94±0.08 (15.74)	2.3±0.12 (27.4)	3.41±0.22(25.8)
96	0.03 (15.01)	NE (14.8)	1.32±0.05 (28.7)	1.6±.14 (27.4)
120	NE (14.87)	NE (13.1)	NE (28.4)	NE (27.1)
168	NE (13.23)	NE (11.26)	NE (27.4)	NE (26.3)

Data is presented as the mean concentration of linezolid released following immersion of wires (n = 6) in PBS. Values in parentheses represent the total drug released till that point and values outside bracket represent the total amount of fresh drug eluted till that time point.

NE: no further elution Error bars represent S.D.

In case of 4% HPMC gel coated wires, a more continuous drug release was seen till a period of 96 h. However, maximum drug was released during the first 30 minutes of immersion (>8 µg/ml). After this, steady release of linezolid (1–2 µg/ml) could be demonstrated after every 2 h till the 8th hour of sampling. By 24 h, additional 6 µg/ml of linezolid was eluted from the coated gel matrix. The total amount of eluted drug at 24 h was approximately 25 µg/ml, though elution continued till 96 h. No further elution occurred beyond this time point and drug concentration remained stable (with a minimal decrease observed) till 168 h of sampling. Hence, 4% HPMC gel that released comparatively higher amount of linezolid at each time point till 96 h was selected for the coating of the wires.

### Phage stability

Phage stability (1.5×10^9^ PFU/ml) was studied in 4% (w/v) HPMC gel as well as on HPMC coated wires on different days. The phage titer showed reduction of one log cycle in the first 24 h in HPMC gel. On day 3, the phage titer dropped further by 1 log cycle. However, it got stabilized thereafter, till day 10 and showed slight decrease in its titer (6.6±2.5×10^6^) by day 20.

The phage on the coated wires also showed uniform stability (Table 3). Although 1.5×10^9^ PFU/ml was used initially for coating, but only 10^6^ phage particles could be coated on the wires. But the number of phage particles eluted from the coated surface remained almost constant throughout the period. Only a minimal drop of 1 log cycle from 2.7±1.5×10^6^ on day 1 to 1.1±1.0×10^5^ PFU/ml was observed on day 20.

**Table 3:** Stability of phage in 4% (w/v) HPMC gel as well as on HPMC coated K-wires (n = 4 per day) on different days.

Days	Phage titer (PFU/ml) in HPMC gel (4%w/v)	Phage titer (PFU/ml) in HPMC + Phage coated wires
1	(2.5±1.1) ×10^8^	(2.7±1.5) ×10^6^
3	(7.25±1.4) ×10^7^	(1.5±2.1) ×10^6^
5	(3.3±2.1) ×10^7^	(7.5±1.0) ×10^5^
10	(1.24±1.47) ×10^7^	(5.2±1.1) ×10^5^
15	(6.89±4.7) ×10^6^	(3.5±2.5) ×10^5^
20	(6.6±2.5) ×10^6^	(1.1±1.0) ×10^5^

Each value represents mean ± S.D of four independent values.

### Total Bacterial adhesion on K-wire*s*

**Figure 1:**
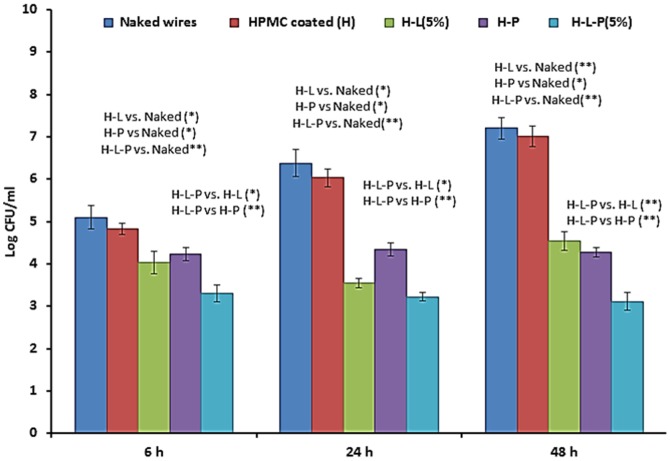
Total biomass of *S.aureus* ATCC 43300 (in terms of Log CFU/ml) adhering to either naked as well as HPMC coated K-wires (n = 4 per group per time point]. Error bars represent S.D. p values among groups have been determined where (*) represent p<0.05 and (**) represent p<0.01.

Naked and coated wires were immersed in bacterial suspension of *S.aureus* 43300 (10^6^ CFU/ml) and taken out (n = 4 per group) at 6 h, 24 h and 48 h. Total viable bacterial cells adhered on each wire were measured in terms of log CFU/ml (Fig. 1).

Initial load of 5.1±0.27 log CFU/ml was observed at 6 h post immersion, which further increased with time giving a maximum load of 7.20±0.25 log CFU/ml at 48 h of immersion. K-wires coated with HPMC only, showed comparatively less adherence with a maximum count of 6.03±0.21 and 7.01±0.24 log CFU/ml at 24 and 48 h respectively. This corresponded to a decrease of 5.5% at 24 h and 2.6% decrease at 48 h. However, this difference was not statistically significant (p>0.05).In case of H-L wires (with 5% [w/w] linezolid) a significant decrease in comparison to naked wire was observed in the first 6 h of immersion which continued during the entire time period. A significant reduction of 2.6 log cycles (p<0.05) in the adhered biomass was seen at 24 h in comparison to naked wires. Maximum adherence of 4.53±0.22 log CFU/ml was seen at 48 h which was equivalent to a significant decrease of 37% in comparison to naked wire. Similarly, in case of phage coated (H-P) wires, the phage was able to significantly reduce the number of adhered cells in the first few hours of immersion. A total bacterial load of 4.23±0.16 log CFU was obtained at 6 h and no further rise in adherence was observed thereafter. A significant reduction of 40% (∼3 log reduction relative to naked wire) was obtained at 48 h when the phage was suspended in HPMC gel. This difference was statistically significant (p<0.05) in comparison to naked wires as well as HPMC coated wires. However, following combination treatment (linezolid [5%] + phage suspended in HPMC i.e H-L-P), a marked reduction in adherence was obtained at all time points. Bacterial adherence on the coated K-wires was prevented at the initial stage itself with maximum load not exceeding beyond 3.51 log CFU. Highly significant reduction of 3.16 and 4 log cycles at 24 and 48 h of immersion (p<0.01) was observed in comparison to both naked as well as HPMC coated wires. The bacterial adherence observed on H-L-P wires when compared to bacterial adherence seen on H-L and H-P wires, was significantly less (p<0.01) at all time points (6, 24 and 48 h).

### Fluorescence microscopy

**Figure 2:**
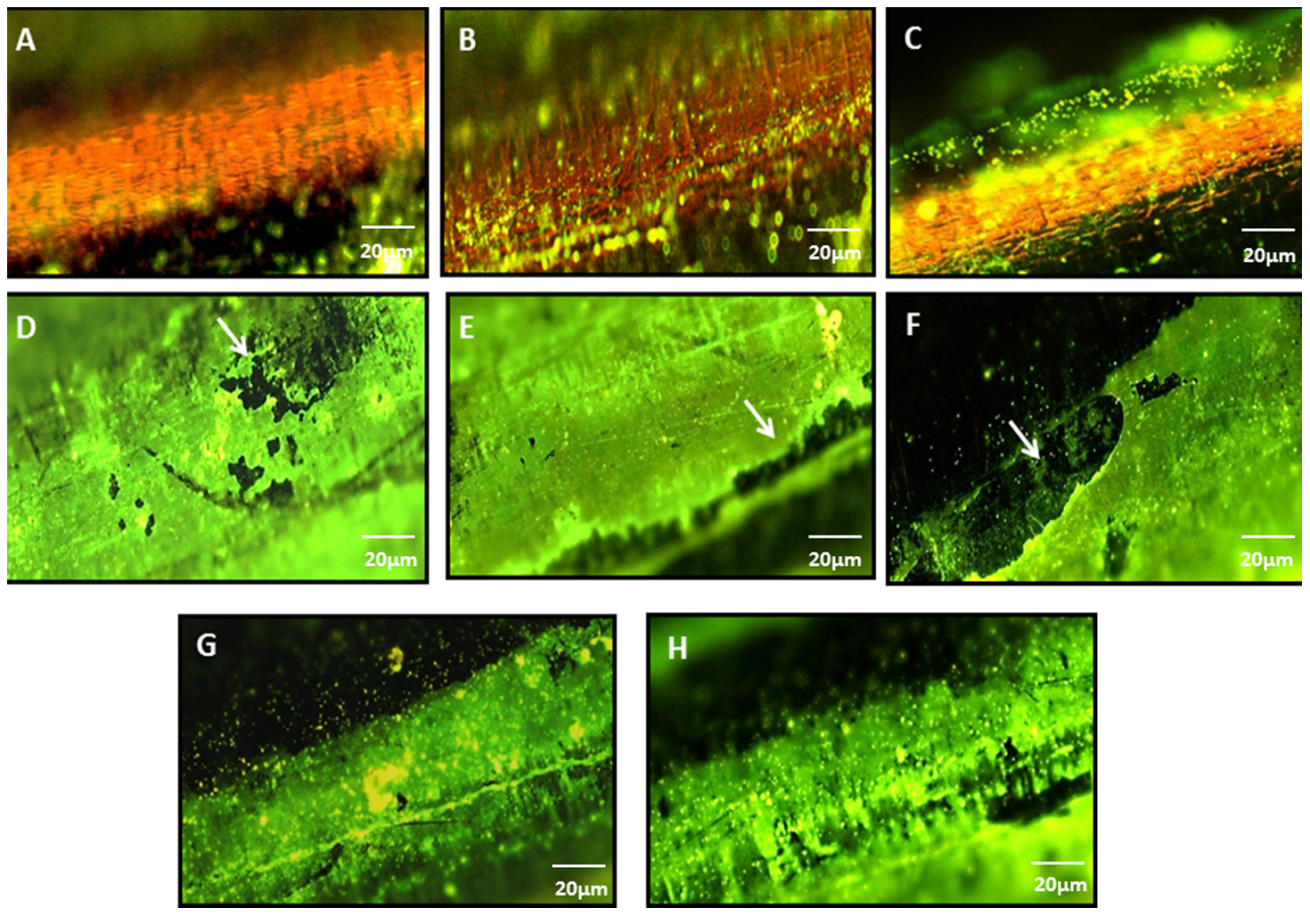
A) Naked wire [not immersed in bacterial suspension] stained with fluorescent dye as viewed under 10x,Nikon eclipse 80i B) naked wire as seen with adhered bacteria (immersed for 6 h in bacterial susupension,10^7^ CFU/ml) C) naked wire as seen with adhered bacteria (immersed for 24 h in bacterial susupension,10^7^ CFU/ml) D) and E) Syto 9 stained HPMC coated wires (not immersed in bacterial suspension) showing coating on the wire F) and G) HPMC coated wires (immersed for 6 h in bacterial susupension,10^7^ CFU/ml) as seen with adhered bacteria (seen as green spots) and H) HPMC coated wires as seen with adhered bacteria (immersed for 24 h in bacterial susupension,10^7^ CFU/ml). (Note: Although the coating was uniform but the white arrows show slight discrepancy in the coating at some locations on wires)

The purpose of staining the K-wires was to visualize the adhered bacteria on the surface of the implants. Hence, naked as well as coated wires (with adhered bacteria) were stained with Syto9 (for live cells) and propidium iodide (stains dead cells) using the Bac-Light Viability kit. In addition, naked wire (Fig. 2A) and HPMC control wires (Fig. 2D) not exposed to bacteria were also visualized. It was observed that wires coated with the polymer (HPMC) alone and not exposed to bacterial suspension evenly took up the green stain i.e Syto 9.Although this precluded us to clearly visualize the adhered cocci on the stained K-wires, but the green background clearly indicated the evenness and uniformity of the HPMC coating(Fig. 2D–2G).Naked wires without the coating did not take up any stain and the adhered bacteria could be seen as tiny green spots (10x, Fig 2B and 2C) after 6 and 24 h of immersion in bacterial suspension. Fig. 2F–H show adhered bacteria on HPMC coated wires following 6 h and 24 h post-immersion.

**Figure 3:**
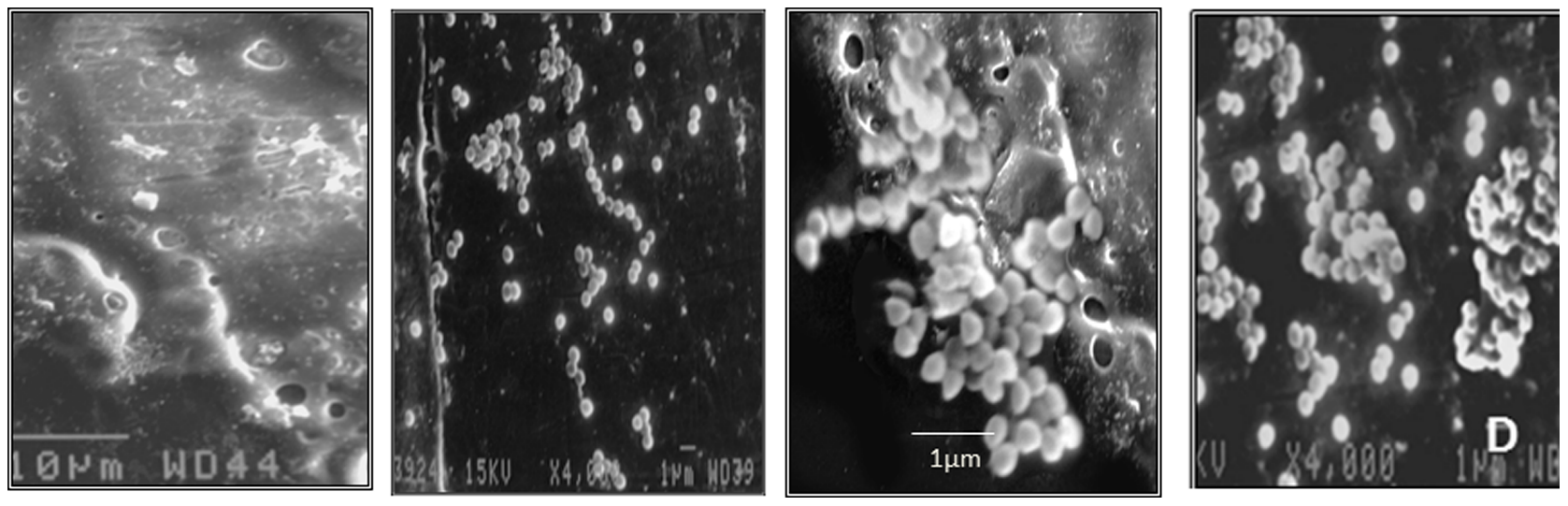
A) HPMC coating as viewed under x1200 B) Initial adherence of *S.aureus* cocci seen after 6 h of immersion on coated surface C) Magnified view of cocci adhered (formation of microcolonies) as bunches on the interface of coated and uncoated surface after 24 h of immersion and D) View of adhered bacteria on coated K-wire as seen after 24 h of immersion.

Polymer coating with 4% HPMC was also viewed under scanning electron microscope (Fig. 3). A uniform coating was seen (Fig. 3A) with slight imperfections, possibly due to lack of appropriate drying at the sample preparation step itself. Initiation of adherence with *S.aureus* 43300 on coated K-wires was seen at 6 h (Fig. 3B) that led to the formation of microcolonies by 24 h (Fig. 3C and 3D).

### Spontaneous frequency of resistance

The frequency of emergence of resistant colonies using linezolid was low. The frequency of the *in vitro* appearance of linezolid resistant mutants was 5×10^−9^. The calculated bacteriophage insensitive mutant (BIM) frequency at multiplicity of infection (MOI) of 10 was comparatively higher with a value of 1×10^−7^.However, when both the agents were used in combination as shown in Table 4, mutation rate was below the detection limit (<10^−9^).The results clearly depict the effectiveness of combination treatment in decreasing the frequency of mutant generation.

**Table 4:** Mutation frequencies to resistance for linezolid and phage in *S. aureus* 43300 (MRSA).

Phage (MR-5) MOI-1*	Phage (MR-5) MOI-10**	Linezolid at (8 µg/ml)	Phage + linezolid (MOI-1+8 µg/ml)	Phage + linezolid (MOI-10+8 µg/ml)
(7.5±1.1) ×10^−6^	(1±0.31) ×10^−7^	(5±1.2) ×10^−9^	<10^−9^	<10^−9^

MOI-1*: phage added at a multiplicity of 1 i.e 10^9^ PFU of phage added.

MOI-10**: phage added at a multiplicity of 10 i.e 10^10^ PFU of phage added.

## Discussion

Coating of medical implants with polymers which have inherent antibacterial activity or loading of polymers with antimicrobial agents is an attractive option to prevent initial attachment and colonisation of bacteria on the implant. These local drug-delivery systems ensure availability of antibiotics at higher concentration around the implant, thereby restricting the biofilm formation by the infecting organism. This strategy also protects the host from the risks and side-effects of long term systemic drug application [3], [43], [47]. In the present study, the efficacy of a new antibacterial surface coating using biodegradable dual delivery system has been investigated. The dual agents used included a potent MRSA antibiotic i.e linezolid that has never been studied as a local delivery agent against orthopaedic implant infections) along with a strongly lytic self-propagating bacteriophage MR-5; covering a broad spectrum of both MSSA as well as MRSA strains (standard and clinical strains). The aim was to prevent adherence and thus colonization of resistant strain of *S.aureus* on orthopaedic implants i.e K-wires by delivery of phage and drug locally at the site of action. This approach can be considered novel as none of the workers earlier have studied the use of lytic phage in combination with antibiotic for their local delivery in the affected bone tissue. This dual delivery system offers some unique advantages due to the inherent merits associated with both the agents. Firstly, phages are capable of self-multiplying locally leading to presence of phages at higher concentration around the implant and diffusing in the surrounding areas till its bacterial host is present at the site. This overcomes the biggest drawback of antibiotic based delivery systems whereby the concentration of antibiotic keeps on decreasing to sub-therapeutic levels from the time of its release. Secondly, the other agent i.e linezolid has a major advantage that it lacks inherent cross-resistance to other antibiotic classes that are given systemically to patients after orthopaedic surgery. In addition it also itself possesses low potential of developing intrinsic resistance [48]–[50].

Phage MR-5, a non-enveloped dsDNA broad spectrum bacteriophage belonging to *Myoviridae* family, was selected for the present study. This is a lytic phage and such lytic phages are always safe and favoured to be used in phage therapy. They do not integrate their genetic material in the bacterial chromosome upon infection, thereby reducing the possibility of transduction of virulence or resistance genes [51], [52].

The phage was mixed with HPMC gel of similar viscosity (K4MP, 4000cps) and used for coating the K-wires. HPMC is a known release modulator with excellent biocompatibility [53]–[55].Three different concentrations of HPMC polymer were used for coating the K-wires. The primary aim was to select the polymer concentration giving a steady and prolonged release of phage and linezolid from the wire surface.

It is known that polymer concentration plays an important role in the release of incorporated agents from the gel matrix [56].While studying the phage elution profile, it was observed that 2% HPMC gel showed an immediate release of phages into the PBS solution with 50% release in the first hour. However, a gel with higher HPMC content (4%) showed slow and steady release of phages upto a period of 96 hours. A prolonged release is more desirable than immediate release of the antibacterial agent as it will be effective over a longer period of time. This difference in the release profile of phage is clearly due to the differences in polymer (HPMC) concentration. Higher polymer concentration results in higher viscosity of the formed gel. Increased viscosity of the HPMC gel makes the gel layer more resistant to dilution and erosion, thus slowing the release of the incorporated phages from the gel matrix. Moreover, thicker polymer gels results in maximum entrapment of more phage particles, leading to the elution at a higher concentration of phages being eluted over a longer period of time. A similar observation has been made by previous workers who have demonstrated that sustained drug release can be achieved by increasing the polymer concentration and higher HPMC content reduces the drug release rate [53], [57]. Though, a significant loss in phage titer was observed at the coating step, yet, the amount of phages released (10^6^ PFU/ml) was enough to infect its host and once inside host phages can easily multiply, reaching titers high enough to tackle the pathogen as long as it was present at the site.

A similar trend was observed while studying the elution profile of linezolid using different polymer concentrations. Gel prepared with 4% polymer gel showed comparatively high amount of drug release at each time point unlike the gel with lower polymer content (2% gel). This might be due to the fact that 4% HPMC gave a more dense coating on the wire, leading to higher concentration of linezolid being trapped within the gel matrix. Although maximum drug was eluted during the first 30 minutes of immersion (>8 µg/ml) but a steady release of linezolid from the matrix was noticed thereafter. The total drug released at 96 h was 28 µg/ml with no change in its concentration on further incubation. This concentration of linezolid was more than 10-fold higher than the determined MIC value (2 µg/ml) of linezolid for *S.aureus* 43300 (MRSA). Workers in the past have reported that although linezolid given orally rapidly reaches the infected tissue compartments of joints and tissues surrounding the bone, in concentrations greater than twice the MIC but the intra-bone tissue concentration was always less. This was demonstrated in a study where the linezolid concentration was found to be below the MIC for 90% of the strains tested [33].However, on the basis of the observation made in this study, local administration by this approach will ensure delivery of linezolid at concentrations, much higher than the MIC level.

Phage stability was studied in 4% HPMC gel formulation as well as on coated wires for a period of 20 days. In both the situations, it was found to be stable with a minimal loss of 1 log cycle in phage titer. HPMC has been found to be highly compatible with bacteriophages as demonstrated earlier. Kumari *et al.* [40] showed 100% stability of Kpn5 phage in 3% HPMC hydrogel preparation over a 7 day period and used it for the treatment of burn wound in mice. Similarly, Alfadhel *et al.* [42] also demonstrated no loss of lytic activity while mixing high titer of phages with HPMC. The latter has been shown to exert better stabilizing effect on the phage as compared to PEG 6000 [58].These observations confer that such high molecular weight polyol polymers can serve as useful formulation excipients for bacteriophages.

Naked wires showed maximum adherence of 6.38±0.33 and 7.20±0.25 log CFU/ml after 24 and 48 h of immersion. The SEM images show a magnified view of cocci adhering onto the interface of coated and uncoated surface in bunches. These cocci are capable of initiating the process of microcolony formation on the K-wires within 24 h of attachment. McMillan *et al.* [9] also demonstrated a high bacterial load on uncoated K-wires on exposure to *S.aureus* Xen 29 (10^6^ CFU/ml).Although, HPMC coated wires showed uniform reduction in bacterial adherence at all time points yet this decrease was not statistically significant (p>0.05).On the contrary phage impregnated in HPMC gel, showed a significant reduction of ∼3 log cycles in bacterial adherence. The phage got eluted in the first 30 minutes in PBS, reaching a peak concentration of 10^6^ PFU/ml within 24 h. Since phages possess the ability of replication at the cost of host bacteria, hence they become available in abundance at the desired site [23].This study demonstrates that owing to their self- replicating nature, the phage MR-5 did not allow any further increase in adherence of *S.aureus* 43300 on K-wire surface. Similarly, linezolid (5% [w/w]) also successfully decreased the bacterial adherence by 2.6 log cycles (44% reduction) on the coated wire in comparison to the naked wire (p<0.05).

A combination therapy with antimicrobials differing in their mechanisms of action has been suggested to treat infections. This approach not only provides a broad spectrum of action due to synergistic effect but it also helps in preventing the emergence of drug-resistant subpopulation. It has been proposed that bacteria acquiring simultaneous resistance to both the phage and antibiotic is remote [59], [60]. Moreover, for combination therapy, the dose of the antimicrobial agent used can be lower than when used singly [61].Linezolid being a synthetic molecule itself possess low intrinsic resistance potential. The results of this study suggest that when used in combination with phage, frequency of emergence of spontaneous mutants was effectively decreased to negligible levels as it was even less than 10^−9^.

In the present manuscript, the efficacy of dual therapy using phage and linezolid has been assessed against standard MRSA strain. However, the same approach shall also work equally well in treating implant infections caused by MSSA strains which are more prevalent in many areas. This approach is superior over using the two agents alone as the decrease in bacterial adherence brought by combined coating strategy was much higher (p<0.01) than using a single agent alone. The minimal bacterial load adhered on such coated K-wires can be taken care by the systemic therapy given immediately after arthroplasty surgery. In addition, the emergence of resistant mutants, that often result in treatment failures and relapse, was significantly reduced beyond the detection limits when exposed to both the agents. Further, *in vivo* evaluation of this approach needs to be investigated and the work in this direction is in progress in our laboratory.
